# Patterns and drivers of climatic niche dynamics during biological invasions of island‐endemic amphibians, reptiles, and birds

**DOI:** 10.1111/gcb.16849

**Published:** 2023-07-03

**Authors:** Adrián García‐Rodríguez, Bernd Lenzner, Clara Marino, Chunlong Liu, Julián A. Velasco, Céline Bellard, Jonathan M. Jeschke, Hanno Seebens, Franz Essl

**Affiliations:** ^1^ Division of BioInvasions, Global Change and Macroecology, Department of Botany and Biodiversity Research University of Vienna Vienna Austria; ^2^ Université Paris‐Saclay, CNRS, AgroParisTech, Ecologie Systématique Evolution Gif‐sur‐Yvette France; ^3^ College of Fisheries Ocean University of China Qingdao China; ^4^ Institute of Hydrobiology Chinese Academy of Sciences Wuhan China; ^5^ Instituto de Ciencias de la Atmósfera y Cambio Climático Universidad Nacional Autónoma de México Mexico City Mexico; ^6^ Institute of Biology Freie Universität Berlin Berlin Germany; ^7^ Leibniz Institute of Freshwater Ecology and Inland Fisheries (IGB) Berlin Germany; ^8^ Berlin‐Brandenburg Institute of Advanced Biodiversity Research (BBIB) Berlin Germany; ^9^ Senckenberg Biodiversity and Climate Research Centre Frankfurt Germany

**Keywords:** alien species, climate, islands, niche conservatism, niche margin index, niche shifts, prediction, vertebrates

## Abstract

Shifts between native and alien climatic niches pose a major challenge for predicting biological invasions. This is particularly true for insular species because geophysical barriers could constrain the realization of their fundamental niches, which may lead to underestimates of their invasion potential. To investigate this idea, we estimated the frequency of shifts between native and alien climatic niches and the magnitude of climatic mismatches using 80,148 alien occurrences of 46 endemic insular amphibian, reptile, and bird species. Then, we assessed the influence of nine potential predictors on climatic mismatches across taxa, based on species' characteristics, native range physical characteristics, and alien range properties. We found that climatic mismatch is common during invasions of endemic insular birds and reptiles: 78.3% and 55.1% of their respective alien records occurred outside of the environmental space of species' native climatic niche. In comparison, climatic mismatch was evident for only 16.2% of the amphibian invasions analyzed. Several predictors significantly explained climatic mismatch, and these varied among taxonomic groups. For amphibians, only native range size was associated with climatic mismatch. For reptiles, the magnitude of climatic mismatch was higher for species with narrow native altitudinal ranges, occurring in topographically complex or less remote islands, as well as for species with larger distances between their native and alien ranges. For birds, climatic mismatch was significantly larger for invasions on continents with higher phylogenetic diversity of the recipient community, and when the invader was more evolutionarily distinct. Our findings highlight that apparently common niche shifts of insular species may jeopardize our ability to forecast their potential invasions using correlative methods based on climatic variables. Also, we show which factors provide additional insights on the actual invasion potential of insular endemic amphibians, reptiles, and birds.

## Graphical abstract

Shifts between native and alien climatic niches pose a major challenge when predicting biological invasions. Analyzing data for 46 species of birds, amphibians, and reptiles, we found that this is particularly true for insular species. We show that climatic mismatch is common, mainly during invasions of endemic insular birds and reptiles. In addition, we found that depending on the taxonomic group such a trend can be explained by several variables including native range size, native altitudinal distributions, topographic complexity, distance between native and invaded ranges, phylogenetic diversity of the recipient community, and evolutionary distinctiveness of the alien species.

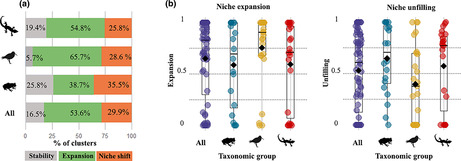

## 1 INTRODUCTION

Biological invasions are an important driver of global environmental change, affecting native biota, ecosystem functioning and services, as well as human livelihoods (Diagne et al., 2021; Pyšek et al., 2020; Vilà et al., 2011). Efforts have been made to improve the understanding of environmental and anthropogenic drivers of biological invasions globally (e.g., Blackburn et al., 2016; Dawson et al., 2017), but our knowledge on the underlying mechanisms is still limited. Despite multiple initiatives to minimize the introduction and spread of alien species, their numbers have grown rapidly during the last decades (Seebens, Blackburn, et al., 2017) and are expected to rise further (Seebens et al., 2021). Thus, improving our understanding of the drivers and mechanisms underlying biological invasions is crucial to accurately predict and pro‐actively reduce their potential impacts.

With the growing availability of ecological data (e.g., species occurrence records and environmental variables), a suite of approaches has become widely used to project species distributions under different contexts, for example, through linking species occurrence to the environmental and geographic space in which they occur based on ecological niche theory (Elith & Graham, 2009; Guisan & Zimmermann, 2000). Considering that species are shifting their distributions at an unprecedented rate in response to global change (Chen et al., 2011; MacLean & Beissinger, 2017), the characterization of climatic niches and their spatio‐temporal dynamics have become even more relevant (Parmesan, 2006). In invasion science, such correlative approaches have been widely applied for predicting alien species' potential distributions to inform conservation decisions (Jiménez‐Valverde et al., 2011; Pearman et al., 2008; Srivastava et al., 2019). However, these approaches rely on basic assumptions that may not apply to biological invasions and hence influence the accuracy of spatial predictions (Bellard et al., 2018; Pili et al., 2020; Yates et al., 2018). For example, the assumption that climatic niches are conserved between native and alien ranges (i.e., niche conservatism hypothesis) is crucial to properly apply correlative approaches such as species distribution modeling and thus accurately predict potentially suitable alien ranges of species (Elith et al., 2010). Yet, this conservatism assumption is not always fulfilled, especially not for recent invasions, which can lead to false estimates of the projected distributions (Early & Sax, 2014; Palaoro et al., 2013).

Whether the climatic niche of a given species may change between its native and alien ranges has become of importance, not only in the fate of global change (Guisan et al., 2014), but also for the general implications it has on how niche‐based predictions are transferable in space for alien species. So far, conflicting evidence has arisen in the literature both supporting and challenging the idea that alien species retain their native climatic niches (NCNs) in the alien range (Broennimann et al., 2007; Early & Sax, 2014; Liu et al., 2020a; Petitpierre et al., 2012). Therefore, a controversial debate on the role of niche conservatism in the context of biological invasions has unfolded. A recent study assessing over 400 alien species concluded that most species conserve their niches during invasion, and that changes in climatic niche in the alien ranges are rare (Liu et al., 2020a). Interestingly, the small set of 21 species for which an expansion was detected were predominantly island‐endemic amphibians and reptiles (Liu et al., 2020a; Stroud, 2021), suggesting that island endemics might be more prone to experience niche shifts during invasion. Yet, it is insufficiently understood which species' attributes make species more prone to exhibit niche shifts in invasions. Attributes of native species range, like its size, if it is constrained by severe dispersal barriers and thus restricted to limited climatic niche space, are assumed to increase range shifts during invasions (Li et al., 2014; Stroud, 2021).

Here, we investigate how common niche expansions and niche shifts occur during the invasion of endemic island amphibians, reptiles, and birds and explore the underlying eco‐evolutionary drivers. We assess the generality of this phenomenon across 118 invasion clusters (i.e., sets of alien occurrences for a given species within a specific country) around the globe for 46 endemic insular tetrapod species from three taxonomic groups (amphibians, birds, and reptiles). For each invasion cluster, we quantified the magnitude of shifts in climatic niche between the native and alien ranges, and subsequently analyzed the role of species characteristics (body mass, native geographic range size, and evolutionary distinctiveness [ED]) as well as descriptors of the native (elevation range, topographic complexity, remoteness) and alien ranges (insularity, distance to native range, phylogenetic diversity [PD] of the recipient community) in explaining the observed climatic niche shifts.

## 2 METHODS

### 2.1 Study species

For a global assessment of niche shifts of insular amphibians, reptiles, and birds, we compiled all documented invasions of island‐endemic amphibians, reptiles, and birds (i.e., we excluded species native to islands if they were also native to continental regions). To obtain our set of endemic insular amphibians, reptiles, and birds with documented invasions, we followed three subsequent steps. First, we identified all amphibians, birds, and reptiles with known established alien populations using two different alien distribution databases: the alien herptiles database by Capinha et al. (2017) for amphibians and reptiles, and the Global Avian Invasion Atlas (GAVIA; Dyer et al., 2017) for birds. Second, we compiled information on their native ranges based on the IUCN Red List data (www.iucnredlist.org) for amphibians, from the Reptile Database (www.reptile‐database.org) for reptiles, and from Birdlife (BirdLife International & Handbook of the Birds of the World, 2020; downloaded in January 2022) for birds. Third, we identified insular endemic species by overlapping native range maps and island delineations from Weigelt et al. (2014). Our final dataset includes 46 tetrapod species (6 amphibians, 21 reptiles, and 19 birds) with native distributions restricted to islands and known alien populations elsewhere.

### 2.2 Occurrence data

For all 46 species, we extracted their global occurrence records from the Global Biodiversity Information Facility (GBIF; www.gbif.org) using the R package rgbif (Chamberlain & Boettiger, 2017). The dataset (https://doi.org/10.15468/dl.b7zndx) was cleaned using the R package CoordinateCleaner (Zizka et al., 2019) to remove erroneous records. We then assigned the native occurrences based on the native range maps for each species, which resulted in 36,636 native occurrences for the 46 species.

Since the occurrences downloaded from GBIF do not contain information whether these represent native or alien populations, we used data from the DASCO workflow (Seebens & Kaplan, 2022) to distinguish between native and alien populations. DASCO combines geolocated occurrences of species available from GBIF with published regional checklists of alien species helping to identify the alien status of the occurrences more precisely. From DASCO's data, we obtained a set of 80,148 alien occurrences for the 46 species. Given that alien species can be introduced multiple times to different regions, their global alien distribution may in fact consist of several disjunct alien ranges. To account for that, we grouped alien occurrences of individual species at the country level, henceforth called “invasion clusters” and defined here as the set of alien occurrences for a given species located within a country. In the cases where the occurrences are located both in mainland and in overseas territories of the same country, we defined more than one cluster for that specific country (e.g., Portugal and the Azores were considered different clusters), as well as in large countries with clusters occurring in well separated regions (e.g., West, and East coasts of USA). We used these invasion clusters as the units of analyses not only to have a better delineation of different introduction events, but also to avoid the inclusion in the analyses of climates from large intermediate geographic areas where the species is not present. Using invasion clusters also allows estimating the region‐specific descriptors tested here as drivers of niche shift during invasion (e.g., the distance between the invasion cluster and the native range, the definition of the recipient communities or the differentiation between invasions in islands and mainland). See Table S1 details on the invasion clusters considered for each species.

### 2.3 Calculating climatic niche shifts during invasions of island endemics

For describing native and alien niches and quantifying shifts between them, we used eight bioclimatic variables related to temperature and precipitation (Table S1) that has been used in previous studies on biological invasion. We obtained the climatic layers from www.worldclim.org at 2.5 arc min resolution (around 5 km at the equator). Based on this information we implemented two analyses that provide complementary approaches to capture different facets of the niche.

#### 2.3.1 Niche dynamic index

**FIGURE 1:**
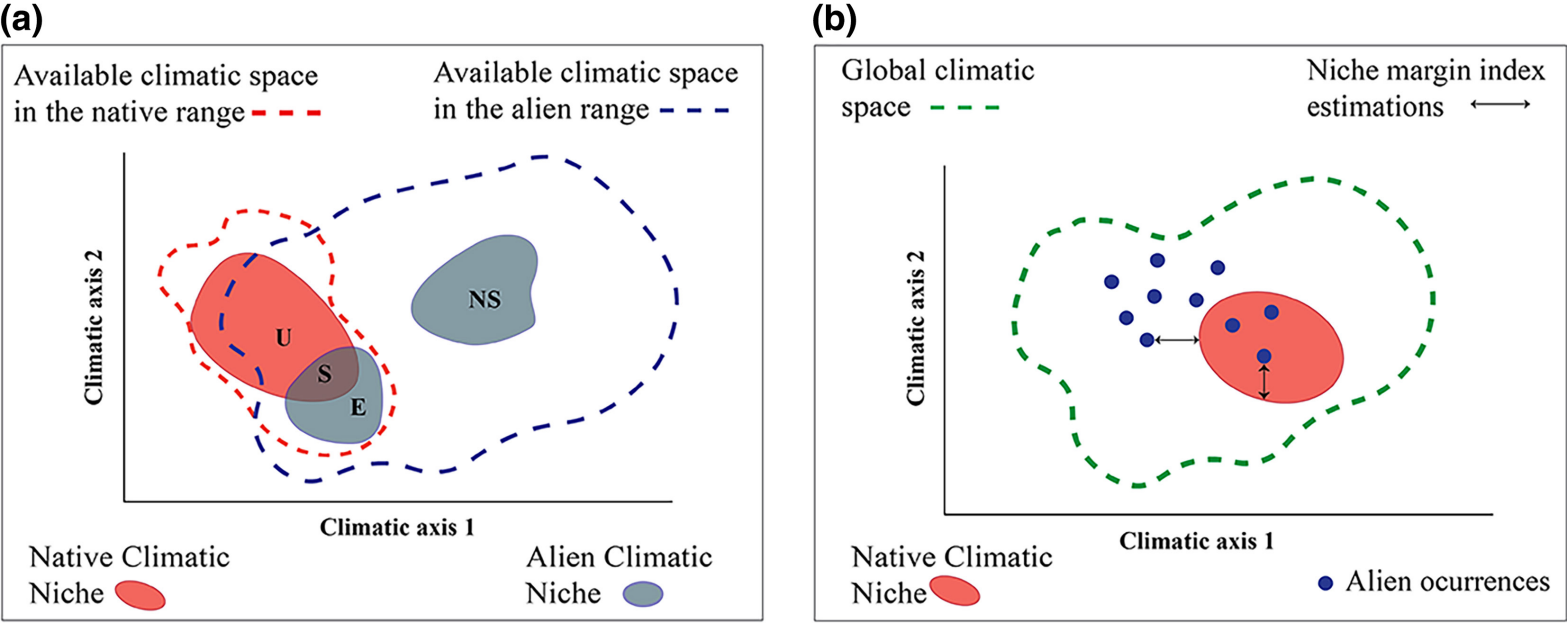
Schematic representation of the two approaches used to quantify niche dynamics during invasions of insular tetrapod species. (a) The COUE scheme estimates overlap based on the construction of two climatic hypervolumes defining the native climatic niche (red polygon) and the alien climatic niche (blue polygon) from the known occurrences in each range. From the overlap of both climatic niches, it is possible to quantify the percentage of climatic space used both in the native and alien range (stability [S]), the conditions occupied in the native but not in the alien range (unfilling [U]), and the conditions occupied by the species only in the alien range. When alien observations occur in analogous climates between native and alien available conditions, we call them expansions (E). In some cases, expansions occur in non‐analogous climates (i.e., climates only available in the alien range); we refer to those changes as niche shifts (NS). (b) The niche margin index (NMI) quantifies the distance (arrows) between each alien occurrence (blue dots) and the nearest margin of the native climatic niche (red polygon) in the global climatic space. If the alien occurrence is located outside the native climatic niche (outerness), the NMI score is negative; and if it is located inside (innerness), the value ranges between 0 and 1, with 1 assigned to occurrences overlapping the species native niche centroid.

To assess climatic niche dynamics during invasion, we applied the COUE scheme (Centroid, Overlap, Unfilling, Expansion; Broennimann et al., 2012; Petitpierre et al., 2012) for each invasion cluster in relation to the species' NCN. COUE is a gold‐standard technique used to test niche conservatism that provides insights on the overall changes in breadth and position between native and alien climatic niches, informing on the levels of niche conservatism and niche shift (Guisan et al., 2014). The COUE scheme uses kernel density smoothers and principal component analysis to summarize environmental conditions in the native and alien ranges in two axes that define a two‐dimensional environmental space for the species (Broennimann et al., 2012). This space is then split into three different components: (i) Stability (S), representing the conditions occupied by the species in both the native and alien range; (ii) unfilling (U), showing the conditions occupied in the native but not in the alien range; and (iii) expansion (E), representing the conditions only occupied in the alien range (Guisan et al., 2014) (Figure 1a). We also quantified niche shifts (NS) as those cases where the alien occurrences fall in non‐analogous climates that are only present in the alien range. We estimated all these metrics with the ecospat R package (di Cola et al., 2017). To test for differences in these metrics between taxonomic groups we ran analyses of variance (ANOVAs). We restricted this analysis to 100 invasion clusters that had five or more occurrences to allow the estimation of the metrics of interest. To explore general trends of niche shifts, we also estimated stability, unfilling, and expansion at the species level, considering unique pools of alien occurrences for each species instead of invasion clusters.

#### 2.3.2 Niche margin index

The niche margin index (NMI; Broennimann et al., 2021) calculates the climatic mismatch between native and alien ranges as the environmental distance between each alien occurrence and its respective species NCN (Figure 1b). This metric quantifies the distance to the NCN margin both inside and outside the hypervolume, and distances are measured as standardized environmental distance between a given site (i.e., each alien occurrence) and the closest margin of the NCN. NMI values range between −∞ and 1, with sites outside the niche having negative values (niche outerness), sites inside the niche having positive values (niche innerness), and zeroes representing sites at the niche margins (Broennimann et al., 2021). According to this scale, a climatic mismatch is higher as the NMI scores become more negative. To estimate the NMI, we used the code provided by Broennimann et al. (2021).

### 2.4 Potential predictors of niche dynamics during invasions of insular tetrapod species

To understand the potential drivers of niche mismatch, we compiled information on nine predictor variables that describe range characteristics, invasion features, and species characteristics. In Table 1, we listed the predictors analyzed and provided a reasoning to justify their inclusion and our expectations for their correlations with climatic mismatch.

**TABLE 1:** Predictors tested as potential drivers of climatic niche shifts during invasions of island endemics.

Type	Driver (proxy)	Rationale	Predictions for niche dynamics during invasion
Native range characteristics	Elevation range	Narrow altitudinal ranges encompass less heterogeneous climates, restricting species access to only a small portion of climatic niche space (Stroud, 2021)	Lower native elevation range results in higher mismatch between native and alien climatic niches
	Topographic complexity (Roughness)	Complex topographies may impose stronger dispersal constraints (Li et al., 2016), resulting in smaller realized portions of the species' fundamental niches	More complex native range topography results in higher mismatch between native and alien climatic niches
	Remoteness	Species from more isolated islands evolve under more disharmonic communities, experiencing less competition (Moser et al., 2018), resulting in larger realized niches.	Species native to less remote islands show a higher mismatch between native and alien climatic niches
Alien range/invasion features	Insularity of alien range	Islands provide less heterogeneous conditions than continental regions (Stroud, 2021), thus chances of finding dissimilar climates during invasion are higher in mainland	Higher mismatch between native and alien climatic niches is expected in invasions of continental regions
	Distance to native range	Distant regions provide more dissimilar conditions than those in the species native range (Seebens, Essl, et al., 2017)	Longer distances between native and alien ranges result in higher mismatch between native and alien climatic niches
	PD of recipient community	Recipient communities with low PD could minimize competition (Elton, 1958), allowing alien species to occupy multiple climatic niches (Ketola et al., 2017)	Lower local PD results in higher mismatch between native and alien climatic niches
Species‐level characteristics	Dispersal ability (Body mass)	Species with good dispersal abilities may occupy a higher portion of their native fundamental niches (this study).	Higher dispersal ability results in lower mismatch between native and alien climatic niches
	PR to recipient community (ED)	The evolutionary uniqueness of an alien species minimizes competition with resident species, facilitating the exploration of novel climatic conditions (Daehler, 2001)	Higher species evolutionary uniqueness means higher mismatch between native and alien climatic niches
	Range size	Alien species with smaller native range sizes experience more truncation of their fundamental niches (this study)	Smaller native ranges result in higher mismatch between native and alien climatic niches

*Note*: The predictors are related to features of the native and alien ranges, but also to intrinsic species characteristics. For each of them, we provide details on the proxy used (when necessary), the rationale supporting its selection and the respective prediction in the context of niche dynamics during invasion.

Abbreviations: ED, evolutionary distinctiveness; PD, phylogenetic diversity; PR, phylogenetic relatedness.

#### 2.4.1 Native range characteristics

To describe species' native ranges, we gathered information on elevation range, topographic complexity, and island remoteness based on the native occurrences. We determined the elevation range in each species' native distribution, by extracting elevation data for all cells within the species range from an elevation layer of the Shuttle Radar Topography Mission (SRTM) at 30 s resolution (available at www.worldclim.org) and subtracted the minimum to the maximum elevation values of the set. We used the R package raster (Hijmans & van Etten, 2010) to calculate roughness, a terrain descriptor of topographic complexity defined as the difference between the maximum and the minimum elevation of a cell and its eight surrounding cells. We first created a global map of roughness using the above‐mentioned elevation layer and then extracted all values for cells falling within the species native range to estimate a mean value of roughness for each species. To calculate island remoteness, we estimated the shortest geographic distance between the native range and the mainland.

#### 2.4.2 Alien range characteristics

To characterize invasion clusters in the alien ranges, we compiled information on (i) the insularity of the alien range (i.e., whether the invasion cluster is located on a continent or on an island), (ii) geographic distances to native range, and (iii) PD of the recipient community. To calculate the distance to the native range, for each invasion cluster we estimated the shortest geographic distance between any of the alien occurrences within a cluster to the native records. For the quantification of PD of the recipient communities, we first created global layers of Faith's PD (Faith, 1992) for each taxonomic group based on the best available phylogenies (for amphibians: Jetz & Pyron, 2018; for reptiles: Tonini et al., 2016 and for birds: Jetz & Fine, 2012) and species geographic distributions available as range maps from IUCN (2022). We estimated Faith's PD using the R package picante (Kembel et al., 2010) and to spatialize the metric created a presence–absence matrix of one geographic degree resolution from the range maps with the R package LetsR (Vilela & Villalobos, 2015). Finally, we used the alien occurrences to extract the respective values from this layer and obtained an average value of PD for each studied invasion cluster. This metric was estimated for each species based on their own taxa.

#### 2.4.3 Species characteristics

For each species, we compiled three variables describing species‐specific characteristics: (i) species native range size, (ii) body mass, and (iii) phylogenetic relatedness to the recipient community (i.e., native species pool within the alien cluster). Both range size and body mass have been related to dispersal ability in several animal taxa (Alzate & Onstein, 2022; Hillman et al., 2014; Sutherland et al., 2000). Given the limited availability of dispersal estimates for most species, we used range size and body mass as proxies for dispersal abilities of species. We calculated species range size by transforming native range polygons from IUCN (2022) to a Mollweide equal‐area projection and then, calculated polygon areas (in km^2^) directly from the native range maps previously compiled using the R package raster (Hijmans & van Etten, 2010). As length is an inadequate measure of body size to compare across taxa with highly variable body shapes (Feldman & Meiri, 2013), we obtained body mass data from different sources, depending on the class. For birds and reptiles, we obtained data for body mass from AVONET (Tobias et al., 2022) and from Slavenko et al. (2016), respectively. For amphibians, we obtained data for snout–vent lengths (SVLs) from AmphiBIO, the global dataset of amphibian traits (Oliveira et al., 2017). In this case, we derived body mass estimates from SVL measurements using length–mass allometric relationships provided by Santini et al. (2018). To determine the degree of phylogenetic relatedness between the alien species and the recipient community, we estimated the alien species' ED score (Isaac et al., 2007), which captures a species' evolutionary uniqueness by measuring the distance along the evolutionary tree from one species (the alien in our case) to its nearest relative (from the pool of species in the recipient community). For each invasion cluster, we pruned the full phylogeny of each group to keep only the species present in each recipient community plus the alien species of interest. We estimated ED scores from these trees using the R package Picante (Kembel et al., 2010). To account for phylogenetic uncertainty, for each cluster we repeated the process across 100 random trees of the posterior distribution of probability of each phylogeny and then obtained a mean value of ED.

#### 2.4.4 Statistical analyses

To identify the role of each predictor for explaining variation in the mismatch between climatic conditions in the native and alien ranges (i.e., NMI as response variable), we ran multiple linear mixed models with a Gaussian error distribution (Bolker et al., 2009). The first model (called “overall model” hereafter) included all taxa. We then ran three subsequent models, one for each tetrapod class separately. All models included the nine explanatory variables as fixed effects and were implemented using the invasion clusters as study units. For the overall model, we included species nested in taxonomic class as a random effect and for the class‐level models, we used species as a random effect. All variables were scaled to mean = 0 and SD = 1 to enable direct comparison of the predictor importance. Models were run using the R package lme4 (Bates et al., 2015). Before running the models, we assessed that predictors were not colinear by estimating Pearson correlations among all of them (Figure S1).

## 3 RESULTS

**FIGURE 2:**
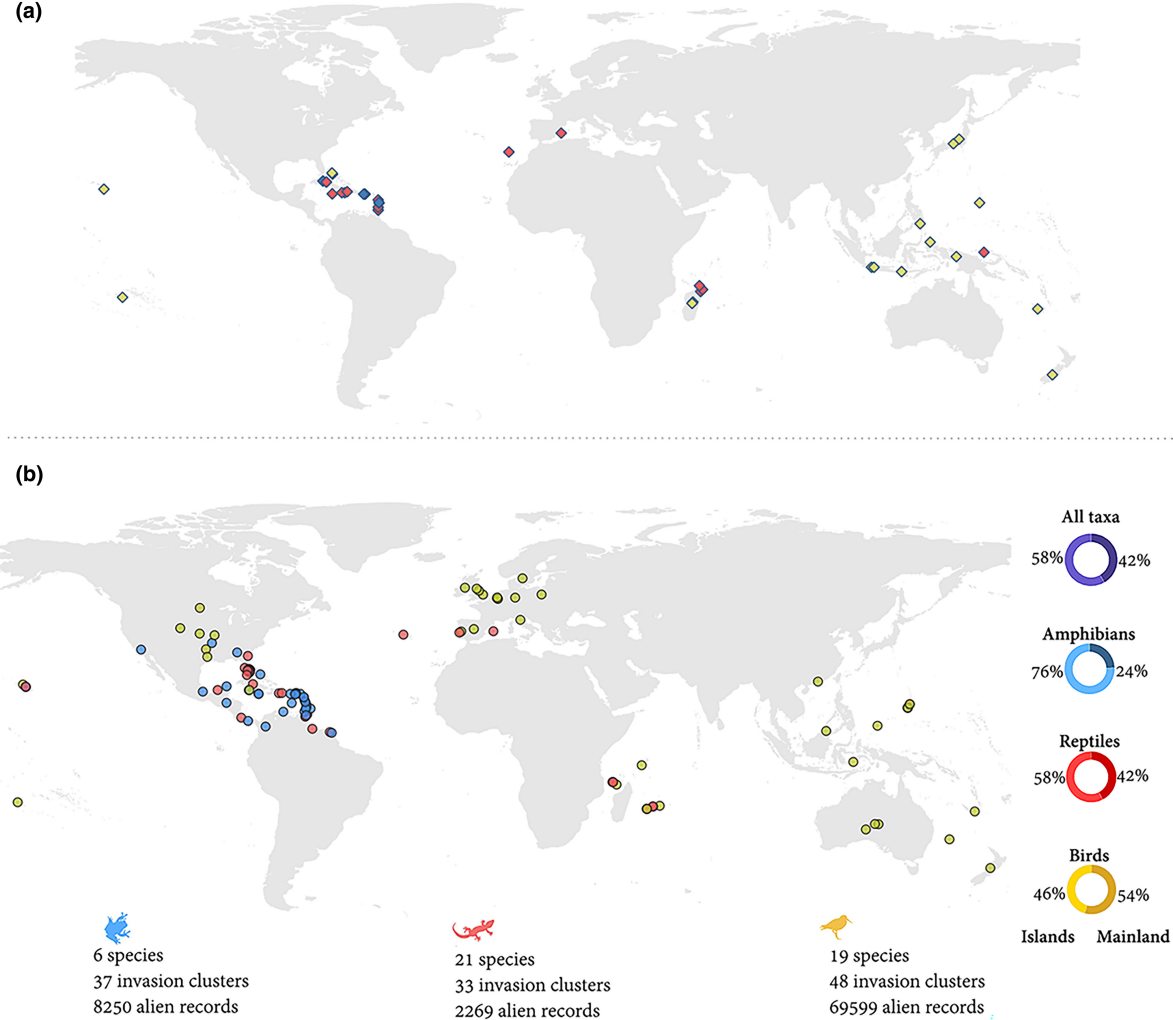
Distribution of centroids for the native ranges and invasion clusters analyzed in this study. The maps show the geographic location of (a) centroids for each species native range and (b) centroids for each of the invasion clusters; in both figures, colors represent the respective vertebrate class. Details of sample sizes for each taxonomic group are also provided at the bottom of the figure. Donut charts at the right in (b) show the proportion of invasion clusters located at the mainland (dark tones) and islands (light tones) in the full dataset and for each taxonomic group.

We found 118 invasion clusters of 46 species spanning 56 different countries around the globe. We considered 37 invasion clusters for amphibians, 33 for reptiles, and 48 for birds (Figure 2).

Invasion clusters analyzed for amphibians are mostly from Caribbean endemics invading other Caribbean islands, the southern USA and Mesoamerica. In reptiles, we evaluated invasion clusters not only in these same regions, but also in Europe, Africa, and the Pacific islands. Bird invasion clusters were evenly distributed across the globe (Figure 2). From the total set of clusters analyzed, 42% were located on islands and 58% in continental regions. In amphibians and reptiles, most invasion clusters occurred more likely on islands (76% and 58% respectively). Contrary, the majority (54%) of bird clusters were located on the mainland (Figure 2; Table S2).

### 3.1 Trends in climatic niche dynamics

**FIGURE 3:**
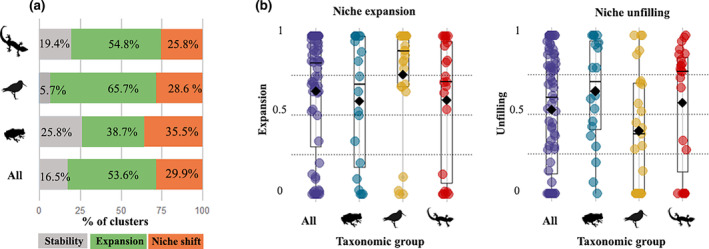
Niche dynamics for the analyzed clusters following the COUE scheme. (a) Percentage of clusters showing conserved niches (high stability), expanded niches (i.e., *E* values higher than 0.5) or niche shifts. (b) Jitter plots showing the distribution of values for niche expansion (left) and niche unfilling (right) obtained for all clusters analyzed (all taxa) and for the clusters belonging to the specific vertebrate classes. In both plots, the black lines inside the boxes represent the median of each dataset and the black diamonds the means. Horizontal dotted lines in (b) are references of the cut‐off values used to define whether expansion and unfilling are weak (*E* < 0.25), moderate (*E* = 0.25–0.5), high (*E* = 0.50–0.75) or very high (*E* > 0.75).

We found a gradient of niche expansion that covers the full scale from no expansion (*E* = 0, *S* = 1) to total expansion (*E* = 1, *S* = 0) across species. In a large proportion (54%) of the clusters analyzed, we found considerable niche expansion (*E* > 0.5). In addition, 30% of clusters presented full niche shifts, where the species occur in the alien range in climates that are not even available in the native region (Figure 3a). These results were consistent with the species‐level estimations of expansion and niche shifts which were detected in 55% and 24% of the species analyzed, respectively (Table S4). Back to the cluster level, full niche shifts were found in 36%, 29%, and 26% of the clusters analyzed for amphibians, birds, and reptiles, respectively (Figure 3a). For the full dataset, we found a mean expansion of 0.65 and a mean unfilling of 0.53. Among taxonomic classes, expansion was higher (*E*_mean_ = 0.76) and unfilling lower on average (*U*_mean_ = 0.39) for birds. For amphibians, we found lower expansion (*E*_mean_ = 0.59) and higher mean unfilling (*U*_mean_ = 0.65) (Figure 3b). Nevertheless, given the high within group variability in these metrics for the three taxonomic classes, one‐way ANOVAs revealed that there are no significant differences among them in terms of niche expansion [*F* (2,94) = 1.112, *p* = .33] and niche unfilling [*F* (2,94) = 1.764, *p* = .177].

### 3.2 Patterns of mismatch between NCN and alien occurrences

**FIGURE 4:**
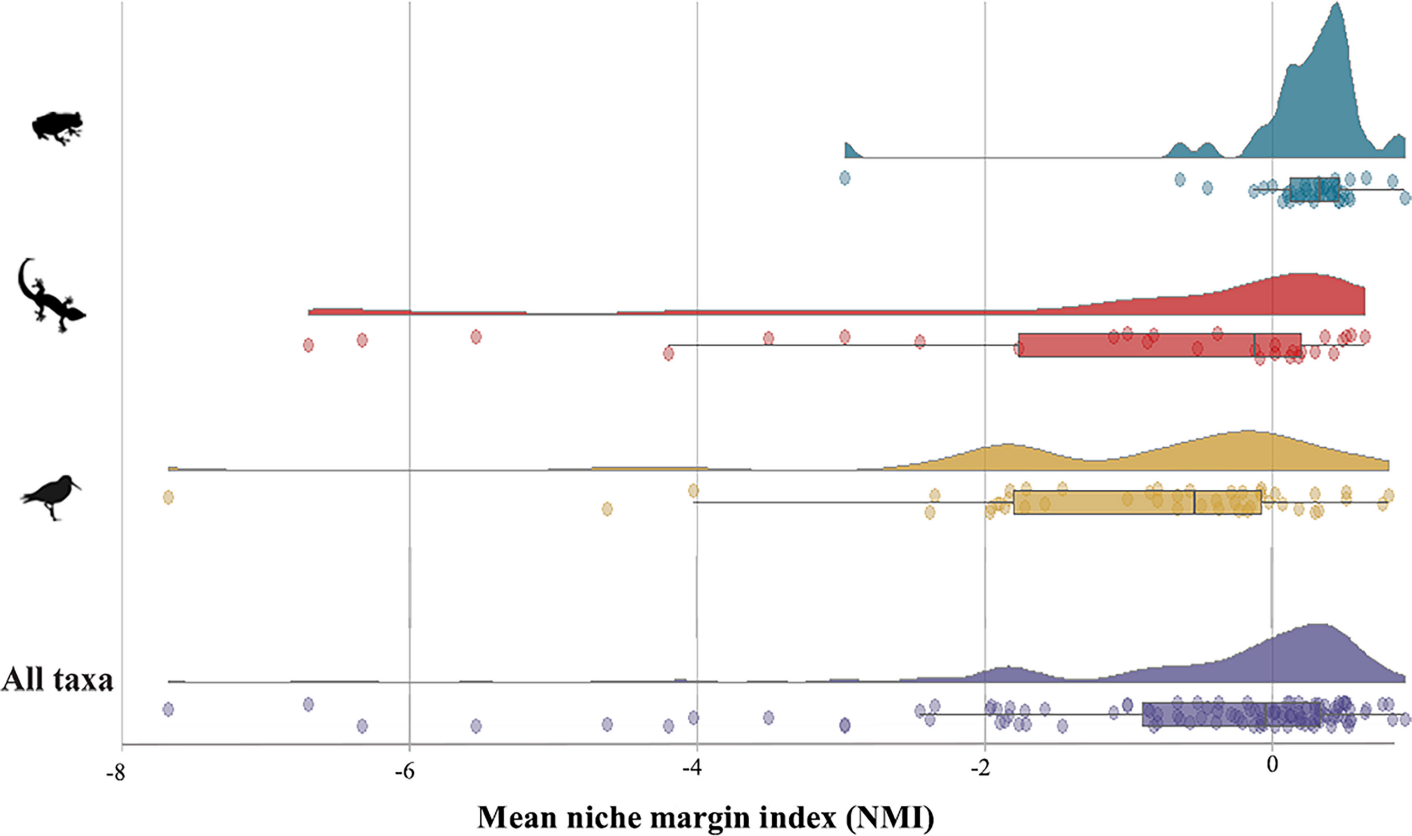
Ridgeline plot showing the distribution of average NMI scores for each of the analyzed invasion clusters. Negative NMI scores correspond to clusters where, on average, alien records occur outside the species' native climatic niche (outerness, higher climatic mismatch); positive NMI scores represent clusters with alien records predominantly falling within the species' native climatic niche (innerness).

From the estimates of the NMI, we found several instances of climatic mismatch in species from the three vertebrate classes analyzed (Figure 4). For 52% of the analyzed invasion clusters, we obtained mean negative values of the NMI, meaning a pronounced climate mismatch between alien and native ranges (Figure 4). The overall mean NMI for all the analyzed invasions was −0.6 ± 1.6. At the class level, average NMI was negative in 16% of the analyzed invasion clusters for amphibians (NMI_mean_ = 0.2 ± 0.6), in 78% of the analyzed invasion clusters for birds (NMI_mean_ = −1 ± 1.5) and 55% of the invasion clusters for reptiles (NMI_mean_ = −1.2 ± 2.1). This indicates that a substantial proportion of alien records of the studied species occurs outside their respective NCN.

### 3.3 Variation in the quantified predictors

The native islands of the analyzed species cover altitudinal gradients as narrow as 45 m (the red‐legged thrush, *Turdus plumbeus*, native to the Bahamas) and as broad as 3600 m (the copper pheasant, *Syrmaticus soemmerringi*, endemic to Japan). Among the native ranges, we quantified the highest terrain roughness (440.26) for the island of Madeira, Portugal, where the lizard *Teira dugesii* is native, whereas the roughness of the Bahamas is hundred times lower (4.39). The most remote (i.e., distance to the nearest continent) species in our dataset are native to French Polynesia (>5000 km), while the least remote occur in the Bahamas (<100 km). Our dataset included both short‐ and long‐distance invasions, varying between a minimum of 26 km and a maximum of 17,877 km between alien and native ranges. The analyzed species varied in body mass between ~1 g (the ocellated gecko, *Sphaerodactylus argus*) and 2000 g (the Hawaiian goose, *Branta sandvicencis*), with native range sizes between 469 km^2^ (*Anolis richardii*) and 1,188,453 km^2^ (*Furcifer oustaleti*). Phylogenetic distance between the alien species and the recipient communities was highest for the invasion of the island thrush, *Turdus poliocephalus* (ED = 0.943), in Australia and lowest for the invasion of the bronze anole lizard, *Anolis aeneus*, in Guyana (ED = 0.002). We also found strong variation in the PD of the recipient communities (PD = 2928.3 ± 2292.3). The full data on the quantified predictors for all species and clusters are available as part of the supplementary material.

### 3.4 Drivers of climatic mismatch in invasions of insular species

**FIGURE 5:**
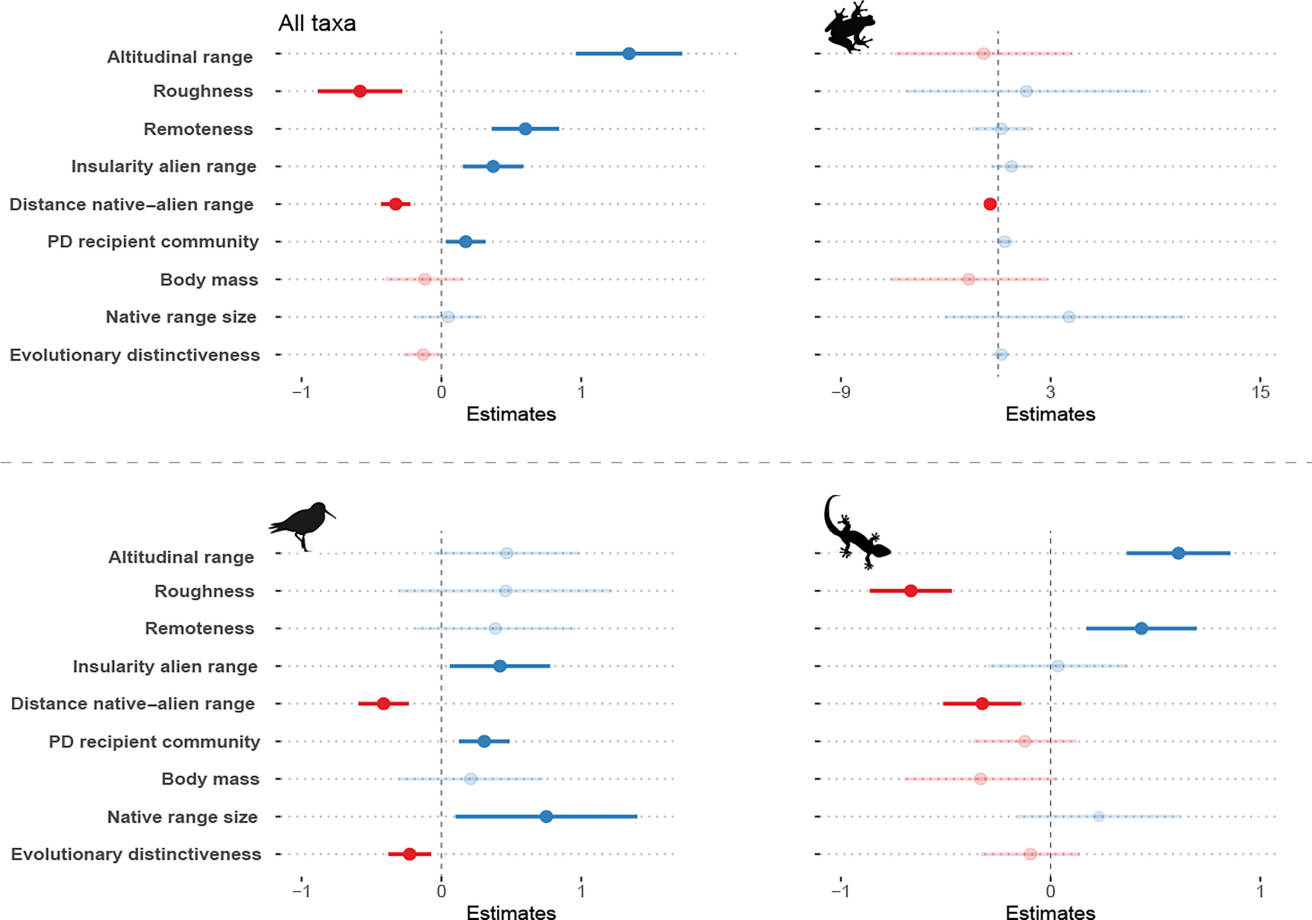
Effect sizes for the nine variables tested on niche margin index as predictors of climatic mismatch between native and alien ranges. Strong red and blue lines show cases where an increase in the value of the variable significantly increases or decreases the climatic mismatch, respectively. Light lines show nonsignificant effects in the models. PD, phylogenetic diversity.

For the full dataset, we found that the degree of climatic mismatch between native and alien occurrences (as estimated by the NMI) increased for species with narrower elevation ranges and from less remote islands, as well as for those with native ranges in highly complex topographies. Climatic mismatch during invasion was also higher in mainland regions than on islands, when the distance between native and alien ranges was large and in recipient communities with low PD (Table 2; Figure 5).

**TABLE 2:** Results from the LMM for the overall model for all three vertebrate classes, considering all predictors and NMI as response variable.

Variable	Estimate	SE	*t*	*p* Value
Altitudinal range	1.36	0.19	7	**<.01**
Topographic complexity	−0.59	0.15	−3.82	**<.01**
Remoteness	0.61	0.12	4.94	**<.01**
Insularity alien range	0.38	0.11	3.4	**<.01**
Distance alien‐native range	−0.33	0.05	−6.15	**<.01**
PD recipient community	0.18	0.07	2.45	**.02**
Body mass	−0.12	0.14	−0.84	.41
Native range size	0.05	0.13	0.4	.69
Evolutionary distinctiveness	−0.14	0.07	−1.82	.07

*Note*: According to this metric, climatic mismatch is higher as more negative the NMI estimates are. Values in bold highlight the variables with significant effects.

Abbreviations: LMM, multiple linear mixed model; NMI, niche margin index; PD, phylogenetic diversity.

For amphibians, only the native–alien distance had a significant, although small, effect following the same relation of increase in climatic mismatch with the increase in distance between native and alien ranges. For birds, we found that climatic mismatch is linked to invasions on islands, invasions in distant regions, recipient communities with low PD, particularly by species with small native ranges and evolutionary distance to the recipient communities. For reptiles, we also found statistical support for increased climatic mismatch in species from less remote islands with complex topographies, narrow elevation ranges and more distance to the alien region (Figure 5; Table S3).

## 4 DISCUSSION

In this study, we show that in many cases insular amphibians, reptiles, and birds undergo niche expansion during invasion of other regions worldwide. We found that a high percentage of the analyzed alien occurrences fall outside the margins of the species' NCN. Moreover, our results show that most of the predictors explain to some degree the mismatch between native and alien climatic niches, however, their importance varies depending on the taxonomic group analyzed. The characteristics of the native region (elevation range, topography, and remoteness) were particularly relevant to predict climatic mismatch in reptile species. For amphibians, however, only the distance between native and alien ranges was significant. For birds, the characteristics of the alien region (i.e., insularity, distance to native range, and PD of the recipient community) and the species intrinsic characteristics (range size and ED) were more informative predictors of climatic mismatch. While we are aware of the drawbacks related to uncertainty, sampling bias, and completeness when using occurrence data to characterize species climatic niches, we were still able to capture multiple examples of climatic mismatch between native and alien niches. This suggests that such a trend, if not general, is at least common among island‐endemic amphibians, reptiles, and birds, meaning that their invasive potential is likely underestimated.

### 4.1 Changes in climatic niches during invasions of insular amphibians, reptiles, and birds

Climate matching has been frequently suggested as an important predictor of invasion success at different stages, from the introduction to the species' successful establishment (e.g., Abellán et al., 2017; Bomford et al., 2009, 2010). However, this assumption has been increasingly challenged (see Bates & Bertelsmeier, 2021, for a comprehensive review). Climatic niche shifts have been documented in several taxa, sometimes corresponding to realizations of different parts of the fundamental niche (realized niche shifts, e.g., Luo et al., 2022), but in other cases representing adaptations to novel climates through evolved environmental tolerance (Huey & Pascual, 2009; Müller‐Schärer et al., 2004). Here, we found instances of one or the other situation in most of the analyzed invasion clusters. In more than half of the analyzed invasion clusters we detected expansions of the realized niche. Remarkably, we also found evidence of niche shifts occurring in non‐analogous climates in 30% of the invasion clusters analyzed. Also interesting is the fact that we found examples of shifts or expansions in five species in our dataset that are categorized as vulnerable, near threatened, or endangered (Table S1). This means that even species that are threatened in their insular native ranges could have an overlooked invasive potential since they are also prone to climatic niche expansions and shifts in the introduced ranges. Our results add to the existing evidence of niche expansions and shifts during invasions, however, comparisons on the prevalence of such cases among studies are hard to set. Indeed, the actual frequency of such events remains unknown due to the subjective interpretations of metrics and the use of dichotomous classifications (Bates & Bertelsmeier, 2021), instead of assessing trends over the continuous gradient between conservatism and shift to which we aimed to adhere here.

Cases of niche shift were most common in amphibians and similarly frequent in birds and reptiles. A poor dispersal ability of amphibians could be thought to restrict their potential to fully fill suitable climatic conditions in the native ranges or limit the tracking of their optimal conditions in the alien ranges; nevertheless, body mass (a proxy of dispersal capacity) did not have any effect on climatic mismatch according to our models. On the other hand, the pronounced shifts found in amphibians may mirror an important capacity of amphibians to adapt to novel conditions, supporting previous evidence that shows their fast rates of niche shifts (Wiens et al., 2019). This could be particularly true for the amphibian species studied here, since most of them are direct‐developing frog species (i.e., species without larval stage; Hedges et al., 2008), less dependent on humid conditions, for example, than other species with tadpole stages during their life cycles.

### 4.2 Characteristics of the native range as drivers of climatic mismatch

The characteristics of the native range have been hypothesized to be important in explaining mismatch due to their influence in the context of niche truncation due to geophysical barriers intrinsic to islands (Li et al., 2014; Stroud, 2021). We found support for this, but only in the case of reptiles, and not for amphibians or birds. Higher climatic mismatch during invasions of reptiles occurs in species from islands with narrow altitudinal ranges. Wide altitudinal ranges provide highly heterogeneous climatic gradients even in short geographic scales (García‐Rodríguez et al., 2021; Rahbek et al., 2019; Suissa et al., 2021). Such climatic heterogeneity is expected to decrease in narrower altitudinal ranges, restricting the environmental space that the species can explore and increasing the likelihood of climatic expansions after introduction in the alien range.

Reptiles from islands with more complex topographies also experience higher climatic mismatch during invasion. Terrain roughness provides a mosaic of hills and valleys that may impose multiple physical barriers for dispersal, increasing isolation and preventing the realization of suitable existing conditions in non‐accessible areas within the native range (Barve et al., 2011). Terrain roughness may be also associated with an increase in habitat heterogeneity and in consequence environmental variability, providing unique native climatic combinations that likely differ from those in the alien ranges. Both conditions may increase the chances for alien occurrences to be situated in novel conditions not previously experienced in the species' native range, that we report here as climatic mismatches during invasion.

We also found that reptile species from less remote islands experience more pronounced climatic mismatch during invasions. This result could be interpreted in two directions. On one hand, it is known that species communities on remote islands are more depauperate and disharmonic compared to less isolated islands, leading to lower competition between species and a higher potential to realize larger parts of their fundamental niches (Moser et al., 2018). Species from less isolated islands, vice versa, are situated in more complex communities with increased competition, likely resulting in a lower realized niche space. However, once in the alien ranges, invasion success could increase in the absence of natural enemies (Heger & Jeschke, 2014; Roy et al., 2011), allowing insular species to explore and occupy additional portions of the climatic space not explored in native conditions, as reflected in our results. In addition, since phylogenetic similarity between communities decays with geographic distance (Morlon et al., 2011), an alternative explanation could be that species from less remote islands may have stronger evolutionary bonds with a wider array of mainland lineages, whether facilitated by shorter dispersal distances or even through past connections to the continents (e.g., as characterized in Weigelt et al., 2014). In the context of niche dynamics, more strongly shared evolutionary histories between the alien species and the recipient community could imply similar environmental tolerances, which would translate into a larger preadaptive potential to deal with novel conditions in the alien range.

### 4.3 Other variables that may drive climatic mismatch

Our study shows that once the species has been introduced to an alien region, other factors related to the alien range and species characteristics may also help to explain the proneness of insular tetrapod species to expand their climatic niches and occur outside the margins of their NCN during invasions. For example, climatic mismatch was higher for birds invading mainlands than islands. This suggests that islands as recipient regions also offer a more restricted climatic space than mainland regions (which in turn are often larger) for the alien species to explore and establish. Thus, the likelihood of finding novel climatic conditions is limited on islands, and the potential for mismatch increases on the mainland. An additional fact to consider is that in our dataset, many bird invasion clusters occur in more temperate continental regions (e.g., Europe) than those of amphibians and reptiles, which itself suppose larger deviations from most of the native ranges studied.

We also found a positive effect (the only one consistent across taxa) of the distance between native and alien ranges, suggesting that independently of species traits, distance may increase the chances for species to occur outside their NCN, even in non‐analogous climates. This effect was stronger in birds and reptiles, weaker in amphibians, likely because most invasion clusters for amphibians are situated in not very distant regions respective to their native ranges. In amphibians in general we did not find major effects of the drivers tested on climatic mismatch. The fact that a lower number of insular amphibians are known to be alien species in relation to the other groups could have prevented us from obtaining more concluding results for this group. Still, we consider it important to study them even when they are not numerous, as the understanding of rare events is particularly relevant in the context of biological invasions.

We directly assessed the role of evolutionary histories of the invader and the native assemblages in shaping the gradients of climatic mismatch by quantifying (i) PD of the recipient community and (ii) ED of the alien species in relation to such communities. Our models show that in the case of birds, climatic mismatch increases in communities with low PD as well as in those cases where the alien species is more evolutionarily distant (high estimates of ED). For the first effect, we argue that recipient communities with low PD may represent evolutionary clusters with likely similar environmental tolerances (Futuyma, 2010; Wiens et al., 2010). As a result of environmental filtering, such species may occupy only a portion of the available climatic space. In terms of climatic mismatch, the unfilled climatic space could provide additional environments for the alien species to use, including those combinations not realized or even nonexistent in their native ranges.

Regarding ED, the influence of evolutionary relatedness on invasions has been widely explored in multiple taxa at the light of two major hypotheses, known together as Darwin's conundrum (Cadotte et al., 2018). On one hand, Darwin's preadaptation hypothesis states that successful invaders tend to be closely related to native species, while the naturalization hypothesis suggests the opposite (Darwin, 1859). The debate of these ideas has been especially intense in recent years due to the emergence of phylogenetic community ecology (Webb et al., 2002). Existing evidence shows partial support for both, highlighting that different processes could be operating depending on the stage and scale of the invasion (Jeschke & Erhard, 2018; Omer et al., 2022; Thuiller et al., 2010). Our results for birds support the naturalization hypothesis, which translated to the context of niche dynamics suppose that when bird invaders are evolutionarily (and likely ecologically) distinct from the native species, they can occur in regions outside their NCN. Contrary to our results, a recent study testing the naturalization hypothesis in birds shows increased invasion success in the presence of close relatives (Sol et al., 2022). More studies teasing apart the role of naturalization and pre‐adaptation during bird invasions are certainly needed to understand its implications on niche dynamics. The evidence supporting the naturalization hypothesis presented here corresponds to the specific case of invasions of island endemics, which represents only a small portion of all documented bird invasions to date worldwide.

In addition to the wide set of drivers tested here, the role of other factors has been evaluated in previous studies dealing with climatic niche dynamics during biological invasions. For instance, it has been observed that, although rare, climatic niche expansions in bird invasions, are more likely during colonization of colder and less seasonal climates (Cardador & Blackburn, 2020). In the same group, evidence for a positive effect of propagule pressure and time since invasion on niche changes has been found (Strubbe et al., 2013). Most studies on this topic were not exclusively focused on island endemics as ours, and the reported frequency of niche expansions was often lower than the one we found. Therefore, there is a need to further explore the recently suggested tendency of island species to change their climatic niches during invasion (Li et al., 2014; Stroud, 2021), which we here confirmed with additional quantitative evidence. Here, we also explored the role of human impact index and time since introduction, but focusing on a small portion of the sample for which such data are available, however, we did not find any significant effects of these variables on climatic mismatch (Figures S2 and S3).

## 5 CONCLUSIONS

Our results show that climatic niche shifts can be frequent during invasions of insular species and support previous findings, challenging the assumption of niche conservatism during invasion for this specific sample of alien species (e.g., Hill et al., 2017; Kumar et al., 2015; Tingley et al., 2014). Most widely used approaches to spatially forecast invasion risk such as species distribution models are correlative and rely on the assumption of niche conservatism (Wiens et al., 2010). Instances of climatic mismatch jeopardize the spatial transferability of such methods (Liu et al., 2020b; Nguyen & Leung, 2022) and in consequence may undermine our ability to forecast the geography of invasions (Early & Sax, 2014; Pili et al., 2020). Our findings show that this is especially true for endemic insular amphibians, reptiles, and birds. These species are likely more strongly limited by dispersal barriers, climatic, and environmental conditions on islands to realize their full environmental niches (Liu et al., 2021; Stroud, 2021). We showed that once introduced to regions outside their native island context, these species are not only released from these constraints but also the combination of characteristics of the alien range as well as the alien species‐specific characteristics may make them more prone to expand their realized niche beyond the native context. While existing methods have their limitations to explicitly anticipate niche shifts, our study shows that compiling information on the native range (e.g., altitudinal range, topographic complexity, and remoteness) as well as on the characteristics of the alien region (e.g., insularity vs. continentality or distance to native range) and the recipient communities can provide useful insights to identify species with masked invasive potential among insular endemics.

## 6 CONFLICT OF INTEREST STATEMENT

All the authors have read this manuscript. We have declared that no competing interests exist and consent to publication of this manuscript.

## Supporting information


Data S1.

## Data Availability

Shapefiles used to delimit the native ranges for the species analyzed are available at the IUCN Red List data (www.iucnredlist.org) for amphibians, at the Reptile Database (www.reptile‐database.org) for reptiles, and at Birdlife (BirdLife International & Handbook of the Birds of the World, 2020), in this last case upon request at http://datazone.birdlife.org/species/requestdis. The delimitation of islands followed here was obtained from Weigelt et al. (2014). The phylogenies used are freely available at www.vertlife.org (Jetz & Fine, 2012; Jetz & Pyron, 2018; Tonini et al., 2016, for amphibians, reptiles, and birds, respectively). Species traits used in this study are freely available from AmphiBIO (Oliveira et al., 2017), AVONET (Tobias et al., 2022), and published databases (Slavenko et al., 2016). Raw occurrences were obtained from GBIF for the species full distributions (https://doi.org/10.15468/dl.b7zndx) and from DASCO (Seebens & Kaplan, 2022) for the alien distributions. The final data and code that support the findings of this study are openly available in Zenodo at https://doi.org/10.5281/zenodo.8059341.
