# Supplementary material for: Patterns and drivers of climatic niche dynamics during biological invasions of island‐endemic amphibians, reptiles, and birds
Source: Glob Chang Biol. 2023 Jul 3;29(17):4924–38. doi: 10.1111/gcb.16849 (PMC10946511; doi:10.1111/gcb.16849)
Supplement: Supplementary file 1 — Data S1. [file GCB-29-4924-s001.docx]

**--Supporting Information--**

**Patterns and drivers of climatic niche dynamics during biological invasions of island-endemic amphibians, reptiles and birds**

**Authors:** Adrián García-Rodríguez, Bernd Lenzner, Clara Marino, Chunlong Liu, Julián A. Velasco, Céline Bellard, Jonathan M. Jeschke, Hanno Seebens and Franz Essl

**Correspondence**

Adrián García-Rodríguez

Division of BioInvasions, Global Change & Macroecology, Department of Botany and Biodiversity Research, University of Vienna, Rennweg 14, 1030, Vienna, Austria

[adrian.garcia@univie.ac.at](mailto:adrian.garcia@univie.ac.at).

**Supplementary tables and figures.**

**Table S1.**  Clusters and number of records analyzed by cluster for the species included in the study.

| Class | Species | Red List Status | Countries with invasion clusters  (No. records analyzed) |
| --- | --- | --- | --- |
|  |  |  |  |
| Amphibia | *Eleutherodactylus antillensis* | LC | Virgin Islands (73), British Virgin Islands (16) |
| Amphibia | *Eleutherodactylus coqui* | LC | Hawaii (203), Virgin Islands (6), USA, West (10), USA, East (6) |
| Amphibia | *Eleutherodactylus johnstonei* | LC | Saint Vincent and the Grenadines (5), Colombia (21), Martinique (31), Jamaica (12), Netherlands Antilles (6), French Guiana (4), Saint Lucia (4), Grenada (5), Barbados (4), Panama (3), Guadeloupe (3), Trinidad & Tobago (3) |
| Amphibia | *Eleutherodactylus martinicensis* | NT | Dominica (20), Antigua and Barbuda (3) |
| Amphibia | *Eleutherodactylus planirostris* | LC | Hawaii (147), Jamaica (6), Honduras (8), USA (1078), Guam (4), Mexico, Yucatan (45), Mexico, Veracruz (5) |
| Amphibia | *Osteopilus septentrionalis* | LC | Bahamas (125), Antigua and Barbuda (6), British Virgin Islands (6), Virgin Islands (56), Cayman Islands (48), Netherlands Antilles (8), USA (6181), Puerto Rico (93), Saint Kitts and Nevis (5), Anguila (3) |
| Aves | *Aerodramus bartschi* | EN | United States (24) |
| Aves | *Branta sandvicensis* | NT | Belgium (9), UK (3) |
| Aves | *Chloropsis cochinchinensis* | EN | Hong Kong (5) |
| Aves | *Corvus moneduloides* | LC | New Caledonia (14) |
| Aves | *Eclectus roratus* | LC | Indonesia (251), Belgium (6), USA (7), Palau (7) |
| Aves | *Foudia madagascariensis* | LC | Mauritius (362), Comoros (28), Mayotte (24), Reunion (368), Seychelles (292) |
| Aves | *Margaroperdix madagarensis* | LC | Reunion (44) |
| Aves | *Ninox novaeseelandiae* | LC | Australia (3123) |
| Aves | *Phasianus versicolor* | LC | Belgium (44), Canada (3), USA (3), UK (3) |
| Aves | *Serinus canaria* | LC | Netherlands (4), Australia (7), Portugal (38), New Zealand (7), Spain (194), England (92), Poland (120), Italy (87), USA (36), Germany (177), Belgium (179), Canada (3), Ireland (5), Midway Islands (24) |
| Aves | *Streptopelia bitorquata* | LC | Northern Mariana Islands (39), Guam (39), Indonesia (3) |
| Aves | *Syrmaticus soemmerringii* | NT | USA (4) |
| Aves | *Tadorna variegate* | LC | Sweden (12), Belgium (9) |
| Aves | *Taeniopygia guttata* | LC | Australia (35565) |
| Aves | *Tanygnathus lucionensis* | NT | Malaysia (118) |
| Aves | *Turdus plumbeus* | LC | Cayman Islands (14), USA (3) |
| Aves | *Turdus poliocephalus* | LC | Australia (3) |
| Aves | *Turnix nigricollis* | LC | Reunion (45), Mauritius (3) |
| Aves | *Vini peruviana* | VU | Cook Islands (22) |
| Reptilia | *Anolis aeneus* | LC | Trinidad and Tobago (30), Guyana (6) |
| Reptilia | *Anolis allisoni* | LC | USA (22) |
| Reptilia | *Anolis chlorocyanus* | LC | USA (19) |
| Reptilia | *Anolis cristatellus* | LC | Dominican Republic (11), Costa Rica (6), USA (193), Dominica (3) |
| Reptilia | *Anolis cybotes* | LC | United States (15) |
| Reptilia | *Anolis garmani* | LC | United States (6) |
| Reptilia | *Anolis marmoratus* | LC | French Guiana (12) |
| Reptilia | *Anolis porcatus* | LC | USA (19), Dominican Republic (5) |
| Reptilia | *Anolis richardii* | LC | Trinidad and Tobago (28) |
| Reptilia | *Anolis trinitatis* | LC | Trinidad and Tobago (8) |
| Reptilia | *Carlia ailanpalai* | LC | Guam (12), Northern Mariana Islands (9) |
| Reptilia | *Furcifer oustaleti* | LC | USA (7) |
| Reptilia | *Furcifer pardalis* | LC | Reunion (68), Mauritius (3) |
| Reptilia | *Leiocephalus carinatus* | LC | USA (728) |
| Reptilia | *Leiocephalus schreibersii* | LC | USA (6) |
| Reptilia | *Phelsuma grandis* | LC | USA (10), Mauritius (9), Reunion (31) |
| Reptilia | *Phelsuma laticauda* | LC | Comoros (8), USA (422) |
| Reptilia | *Podarcis pityusensis* | NT | Spain (10) |
| Reptilia | *Sphaerodactylus argus* | LC | Cuba (7), Mexico (3) |
| Reptilia | *Sphaerodactylus elegans* | LC | United States (18) |
| Reptilia | *Teira dugesii* | LC | Portugal (503), Azores (25) |

**Table S2.** Absolute numbers and respective percentages of analyzed clusters by taxa occurring in islands and mainland.

| Taxa | Continent | % Continent |  | Island | % Island | Total |
| --- | --- | --- | --- | --- | --- | --- |
| Amphibia | 9 | 24.3 |  | 28 | 75.7 | 37 |
| Aves | 26 | 54.2 |  | 22 | 45.8 | 48 |
| Reptilia | 14 | 42.4 |  | 19 | 57.6 | 33 |
| Total | 49 | 41.5 |  | 69 | 58.5 | 118 |

**Table S3.** Results from the LMM for the models for each taxonomic group considering all predictors. Bold fonts and asterisks highlight the variables with significant effects

|  | **Variable** | **Estimate** | **SE** | **t** | ***p-* value** |
| --- | --- | --- | --- | --- | --- |
| **A**  **M**  **P**  **H**  **I**  **B**  **I**  **A**  **N**  **S** | Altitudinal range | -0.83 | 4.84 | -0.17 | 0.86 |
|  | Topographic complexity | 1.61 | 6.25 | 0.26 | 0.8 |
|  | Remoteness | 0.18 | 1.75 | 0.11 | 0.92 |
|  | Insularity alien range | 0.76 | 0.58 | 1.31 | 0.2 |
|  | Distance alien-native range | -0.46 | 0.19 | -2.4 | **0.02*** |
|  | PD recipient community | 0.38 | 0.24 | 1.54 | 0.13 |
|  | Body Mass | -1.68 | 4.07 | -0.41 | 0.68 |
|  | Native range size | 4.05 | 11.13 | 0.36 | 0.72 |
|  | Evolutionary distinctiveness | 0.18 | 0.2 | 0.9 | 0.38 |
|  |  |  |  |  |  |
|  | **Variable** | **Estimate** | **SE** | **t** | ***p-* value** |
| **R**  **E**  **P**  **T**  **I**  **L**  **E**  **S** | Altitudinal range | 0.61 | 0.12 | 5.21 | **<0.01 *** |
|  | Topographic complexity | -0.67 | 0.09 | -7.17 | **<0.01 *** |
|  | Remoteness | 0.43 | 0.12 | 3.48 | **0.01*** |
|  | Insularity alien range | 0.04 | 0.16 | 0.23 | 0.82 |
|  | Distance alien-native range | -0.33 | 0.09 | -3.68 | **<0.01 *** |
|  | PD recipient community | -0.12 | 0.12 | -1.05 | 0.31 |
|  | Body Mass | -0.33 | 0.17 | -1.92 | 0.08 |
|  | Native range size | 0.23 | 0.18 | 1.24 | 0.23 |
|  | Evolutionary distinctiveness | -0.1 | 0.11 | -0.86 | 0.41 |
|  |  |  |  |  |  |
|  | **Variable** | **Estimate** | **SE** | **t** | ***p-* value** |
| **B**  **I**  **R**  **D**  **S** | Altitudinal range | 0.47 | 0.26 | 1.83 | 0.1 |
|  | Topographic complexity | 0.46 | 0.38 | 1.22 | 0.25 |
|  | Remoteness | 0.39 | 0.28 | 1.37 | 0.2 |
|  | Insularity alien range | 0.42 | 0.18 | 2.38 | **0.03*** |
|  | Distance alien-native range | -0.41 | 0.09 | -4.67 | **<0.01 *** |
|  | PD recipient community | 0.31 | 0.09 | 3.45 | **<0.01 *** |
|  | Body Mass | 0.21 | 0.25 | 0.82 | 0.43 |
|  | Native range size | 0.75 | 0.32 | 2.35 | **0.04*** |
|  | Evolutionary distinctiveness | -0.23 | 0.08 | -3.02 | **0.01*** |

**Table S4.** Expansion, stability and unfilling values estimated with the COUE approach at the species level. These results are consistent with the cluster level estimations showing a predominant tendency of niche expansion and even niche shifts during invasions of insular amphibians, reptiles and amphibians.

| Species | Expansion | Stability | Unfilling | Interpretation |
| --- | --- | --- | --- | --- |
| *Aerodramus bartschi* | 0 | 0 | 0 | Shift |
| *Anolis aeneus* | 0.8183 | 0.1817 | 0.1313 | Expansion |
| *Anolis allisoni* | 0 | 0 | 0 | Shift |
| *Anolis chlorocyanus* | 0.2504 | 0.7496 | 0.939 | Stability |
| *Anolis cristatellus* | 0.8966 | 0.1034 | 0.9726 | Expansion |
| *Anolis cybotes* | 0.0702 | 0.9298 | 0.7855 | Stability |
| *Anolis garmani* | 0 | 0 | 0 | Shift |
| *Anolis marmoratus* | 0 | 0 | 0 | Shift |
| *Anolis porcatus* | 0.0219 | 0.9781 | 0.5086 | Stability |
| *Anolis richardii* | 0 | 0 | 1 | Shift |
| *Anolis trinitatis* | 0 | 0 | 0 | Shift |
| *Branta sandvicensis* | 1 | 0 | 0 | Expansion |
| *Carlia ailanpalai* | 0 | 0 | 0 | Shift |
| *Chloropsis cochinchinensis* | 0 | 0 | 0 | Shift |
| *Corvus moneduloides* | 1 | 0 | 0 | Expansion |
| *Eclectus roratus* | 0.6005 | 0.3995 | 0.0092 | Expansion |
| *Eleutherodactylus antillensis* | 0.2084 | 0.7916 | 0.5372 | Stability |
| *Eleutherodactylus coqui* | 0.5073 | 0.4927 | 0 | Expansion |
| *Eleutherodactylus johnstonei* | 0.825 | 0.175 | 0 | Expansion |
| *Eleutherodactylus martinicensis* | 0.9826 | 0.0174 | 0.9802 | Expansion |
| *Eleutherodactylus planirostris* | 0.4576 | 0.5424 | 0.2763 | Stability |
| *Foudia madagascariensis* | 0.884 | 0.116 | 0.9347 | Expansion |
| *Furcifer oustaleti* | 1 | 0 | 1 | Expansion |
| *Furcifer pardalis* | 0.7802 | 0.2198 | 0.51 | Expansion |
| *Leiocephalus carinatus* | 0.5208 | 0.4792 | 0.3817 | Expansion |
| *Leiocephalus schreibersii* | 0 | 0 | 0 | Shift |
| *Margaroperdix madagarensis* | 0.0649 | 0.9351 | 0.2182 | Stability |
| *Ninox novaeseelandiae* | 0.6906 | 0.3094 | 0.4133 | Expansion |
| *Osteopilus septentrionalis* | 0.7081 | 0.2919 | 0.8809 | Expansion |
| *Phasianus versicolor* | 0.9838 | 0.0162 | 0.9942 | Expansion |
| *Phelsuma dubia* | 0.9312 | 0.0688 | 0.9947 | Expansion |
| *Phelsuma grandis* | 0.4185 | 0.5815 | 0.788 | Stability |
| *Phelsuma laticauda* | 0.6728 | 0.3272 | 0.575 | Expansion |
| *Podarcis pityusensis* | 0.76 | 0.24 | 0.8071 | Expansion |
| *Sphaerodactylus argus* | 0.4878 | 0.5122 | 0.9206 | Stability |
| *Sphaerodactylus elegans* | 1 | 0 | 0 | Expansion |
| *Streptopelia bitorquata* | 1 | 0 | 1 | Expansion |
| *Syrmaticus soemmerringii* | 0 | 0 | 1 | Shift |
| *Tadorna variegata* | 1 | 0 | 0 | Expansion |
| *Taeniopygia guttata* | 0 | 0 | 1 | Expansion |
| *Tanygnathus lucionensis* | 0 | 1 | 0.6822 | Stability |
| *Teira dugesii* | 0 | 0 | 1 | Shift |
| *Turdus plumbeus* | 0.5648 | 0.4352 | 0 | Expansion |
| *Turnix nigricollis* | 0.831 | 0.169 | 0.186 | Expansion |
| *Vini peruviana* | 1 | 0 | 0 | Expansion |


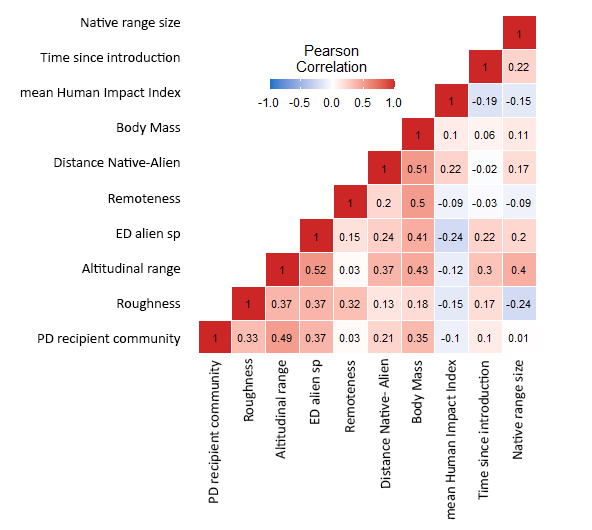


**Figure S1.** Pearson’s correlations among the analyzed drivers.


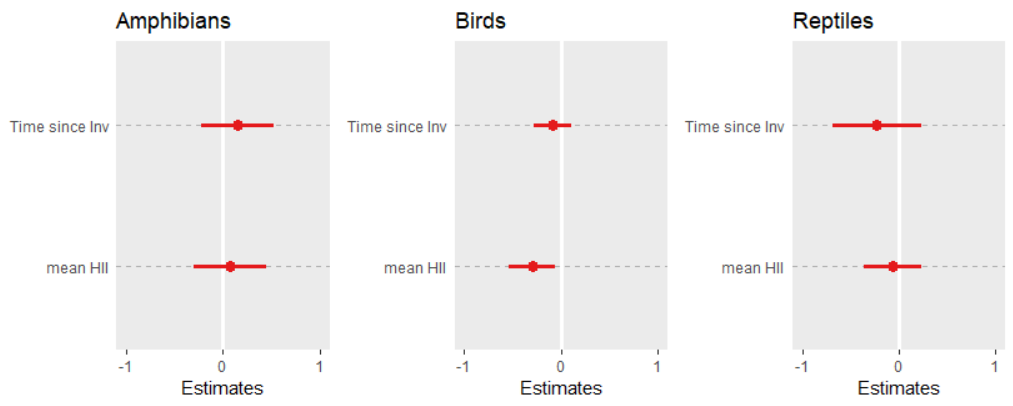


**Figure S2.** Effect sizes of Time since introduction and mean Human Impact Index on the estimations of climatic mismatch in each of the taxonomic groups studied. These models were fitted using a subset of the sample having complete data for both drivers (n=86 clusters) which accounts for around two thirds of the full dataset.


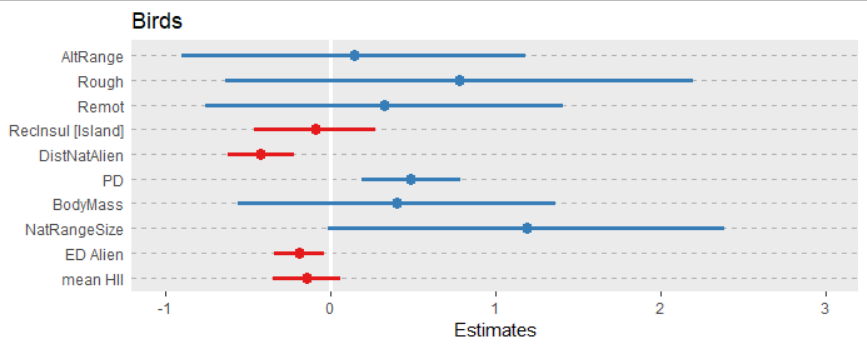


**Figure S3.** Effects of all variables including mean HII on climatic mismatch for birds. We explored further the effect of mean HII as it was significant only for this taxonomic group in the previous analysis, however when considering all variables, the effect turned non-significant.
